# Medical education on hospital hyperglycemia improving knowledge and outcomes

**DOI:** 10.20945/2359-4292-2023-0003

**Published:** 2024-02-19

**Authors:** Jivago da Fonseca Lopes, Pedro da Rocha Andrade, Magno Tauceda Borges, Matheus Carret Krause, Mariano Otto Schmitz Simi, Maristela Bohlke, Leticia Schwerz Weinert

**Affiliations:** 1 Universidade Católica de Pelotas Pelotas RS Brasil Universidade Católica de Pelotas, Pelotas, RS, Brasil; 2 Universidade Federal de Pelotas Pelotas RS Brasil Universidade Federal de Pelotas, Pelotas, RS, Brasil

**Keywords:** Diabetes mellitus, hospital hyperglycemia, insulin therapy, medical education

## Abstract

**Objective:**

To evaluate the effects of medical education on hospital hyperglycemia on physician's technical knowledge and the quality of medical prescriptions, patient care, and clinical outcomes.

**Subjects and methods:**

The intervention included online classes and practical consultations provided by an endocrinologist to medical preceptors and residents of the Department of Internal Medicine. A pretest and a post-test (0 to 10 points) were applied before and after the intervention and patients medical records were reviewed before and after the intervention. The outcomes were improvement in medical knowledge, in the quality of prescriptions for patients in the clinical area, and clinical outcomes.

**Results:**

The global mean of correct answers improved with the intervention [before: 6.9 points (±1.7) versus after the intervention: 8.8 points (±1.5) (p < 0.001)]. The number of patients who did not have at least one blood glucose assessment during the entire hospitalization for acute illness decreased from 12.6% before to 2.6% (p < 0.001) after the intervention. There was also a significant reduction in hospital hypoglycemia rates (p < 0.026). The use of sliding-scale insulin as the main treatment was quite low before and after the intervention (2.2% and 0%). After 6 months, medical knowledge did not show significant reduction.

**Conclusion:**

Medical education on hospital hyperglycemia can improve medical knowledge and clinical outcomes for patients. The improvement in medical knowledge was maintained after 6 months.

## INTRODUCTION

Diabetes is a complex chronic disease that requires ongoing medical care. The disease directly and indirectly relates to the risk of hospitalization and a prolonged hospital stay (1).

Adult patients with diabetes or newly recognized hyperglycemia account for over 30% of non-critically ill hospitalized patients. These patients are at increased risk for adverse clinical outcomes in the absence of defined approaches to glycemic management (2).

Hospital readmissions of patients with diabetes are common and expensive. Major risk factors for readmission include sociodemographics, comorbidities, insulin use, hospital length of stay (LOS), and history of readmissions, most of which are non-modifiable. In retrospective studies and mostly small randomized controlled trials (RCTs), interventions such as inpatient diabetes education, inpatient diabetes management services, transition of care support, and outpatient follow-up generally associate with a reduction in the risk of acute care re-utilization (3). Studies suggest a reduction in LOS and improved clinical care for patients with diabetes after introducing a diabetes inpatient specialist (4). In addition, a meta-analysis shows that introducing a glycemic control protocol that a health team gives, despite having shown to result in a modification of the therapeutic strategy, no changes were observed in glycemic control, frequency of episodes of hypoglycemia and hyperglycemia, or duration of hospitalization (5).

In Brazil, the prevalence of diabetes in the adult population was approximately 7.6% in the late 1980s. More recent data point to a higher prevalence of approximately 20% (6). Regarding hospital admissions, data suggest that 22% of patients hospitalized have diabetes and that hospital admissions accounted for half of the USD 174 billion total medical expenditures for this disease. In the United States, there are 1.6 million new cases of diabetes each year, with a prevalence of 23.6 million people, which is approximately 7.8% of the population, with a quarter of cases remaining undiagnosed (7). The hospital readmission rate in patients with diabetes is between 14% and 20%, especially in the first 30 days after medical discharge (8,9).

The main risk factors related to readmission include lower socioeconomic status, racial group, associated comorbidities, and recent hospitalization (8). Data from medical practice highlight the importance of knowledge about the disease and recognize the intervention of a team specialized in diabetes as an important predictor in the improvement of hospital outcomes, such as hospitalization. However, the scientific literature lacks effective interventions to improve hospital outcomes in patients with hyperglycemia (10,11).

Considering the seriousness of the possible complications of the disease, the increasing costs of treatment, related hospital admissions, high readmission rate, and positive effect of medical knowledge on improving the management of the disease, this study aimed to assess the effect of a medical education program on hospital hyperglycemia in improving medical knowledge and patient outcomes.

## SUBJECTS AND METHODS

### Study design

A "before and after" non-randomized intervention study was conducted in a single center, the School Hospital of Federal University of Pelotas. Physicians included in the study knew of educational activities and their respective pre- and post-test with a medium difficulty level about hospital hyperglycemia; however, physicians and patients were blinded to data collection from patient records. This study was conducted from March 2020 to March 2021.

Hyperglycemia in the hospital was defined by glycemia above 140 mg/dL. Previous diabetes mellitus was defined by patient report or by glycosylated hemoglobin measurement.

### Participants

- Preceptors and residents from medical clinic teams of the present hospital were invited to participate. The same participants answered pre- and post-test. Preceptors and residents from endocrinology were excluded of the study.
- All patients hospitalized in clinical hospital wards 4 months before (March to June 2020) and 4 months after the intervention (August to November 2020) had their medical records reviewed for diabetes diagnosis, treatment, medical prescription and outcomes of interest. Terminal patients from clinical wards were also evaluated. Patients in pediatric, surgical, or gynecological wards were not included.

### Intervention

The preceptors and residents attended a one-hour online theoretical class about hospital hyperglycemia, which a resident in endocrinology and a PhD professor (theorical intervention) taught. After this theoretical class, for one month, the same professionals visited the clinical teams weekly to help manage patients with hyperglycemia, inpatient case discussions, insulin prescription adjustment, clarification of doubts, and hospital hyperglycemia updates based on guidelines in the area (practical intervention). The assistant medical team was the responsible for patient prescription; the specialized team gave theoretical and practical help.

### Outcomes

- Physicians: To assess the improvement in medical knowledge about hospital hyperglycemia, a 10-item questionnaire on the topic was prepared by a specialist in the area and was applied in the pre- and post-intervention periods (Supplementary Appendix 1). After 6 months, the questionnaire was again administered to assess the maintenance of medical knowledge. To assess the reliability of the present instrument, a pilot study was previously conducted by applying the questionnaire to 10 participants. Cronbach’s alpha was 50%, reflecting a moderate level of reliability. To assess the questionnaire’s validity, three endocrinologists assessed the questions regarding necessity, relevance, clarity, and simplicity. The content validity index (CVI) and content validity ratio (CVR) were calculated. The overall CVI, relevance CVI, clarity CVI, and simplicity CVI were 0.934, 0.933, 0.906, and 1.0, respectively. The critical point of 0.75 was selected for the CVR (12). All individual questions had a CVR of 1.
- Patients: To assess the improvement in the quality of care provided to the patient, medical records were reviewed after discharge to investigate the rates of hyper- (>180 mg/dL) and hypoglycemia (<70 mg/dL), of correct prescription of insulin or oral antidiabetics, of glycemic monitoring during hospitalization, of antibiotic therapy use (hospital infection), and the length of stay in the hospital. For the glycemia outcome analysis, we used capilar glycemia measurements of the last 3 days of hospitalization. The hospital readmission rate in 30 days was verified via a telephone call.

### Statistical analysis

Dichotomous variables were described as numbers and percentages, and quantitative variables were described as means, standard deviations, medians, and interquartile ranges. For continuous variables, comparisons were performed using a t-test for gaussian variables and a Wilcoxon test for non-gaussian variables. Chi-square and Fisher’s exact tests were used for qualitative variables.

The significance level adopted was 5%. Database and statistical analyses were performed using the statistical program SPSS Statistics 22 (Statistical Package for Social Sciences – Professional Statistics).

The sample size calculation was based on a previous study on medical knowledge with a rate of a correct answer of 46.44% (13) (average correct answer for the four main questions). Assuming medical knowledge after the intervention approaches 80% of correct answers, a study power of 80% and an alpha error of 5%, it is necessary to evaluate 62 medical questionnaires, with 31 administered before and 31 administered after the intervention.

### Ethical aspects

Physicians had the right to refuse to participate in the study. The pre- and post-test was administered to all medical professionals observing confidentiality and after signing informed consent forms. Data from medical records and other information about hospital data were collected with the institution’s consent after signing the consultation form for confidentiality. The Brazil platform (26087019.6.0000.5317) and the Research Ethics Committee of the Federal University of Pelotas (3.772.481) approved the study on December 15, 2019.

Due to the COVID-19 pandemic, the theoretical class was taught online. Visits to clinical teams and/or clinical specialties were conducted with the appropriate protective equipment.

## RESULTS

A total of 124 medical questionnaires were evaluated (63 before and 61 after the intervention). No doctor refused to participate, but two did not answer the post-test and four professionals were not included because of absence from work. There was no relevant change in the clinical staff of the researched hospital during the follow-up period. Table 1 shows the sample characteristics of physicians.

**Table 1:** Characteristics of the sample of physicians evaluated before and after the hospital hyperglycemia education program

Variables	Before Intervention (n = 63)	After intervention (n = 61)
Women (n)	35 (55.6)	33 (54.1)
Preceptor physicians* (n)	31 (49.2)	29 (47.5)
Resident physicians (n)		
Internal medicine	28 (44.4)	28 (45.9)
Gastroenterology	4 (6.4)	4 (6.6)

Data presented as n (%).

* Distributed among the specialties of medical clinic, nephrology, gastroenterology, intensive care medicine, cardiology, pneumology, oncology, rheumatology, infectious diseases and allergy and immunology.

**Figure 1:**
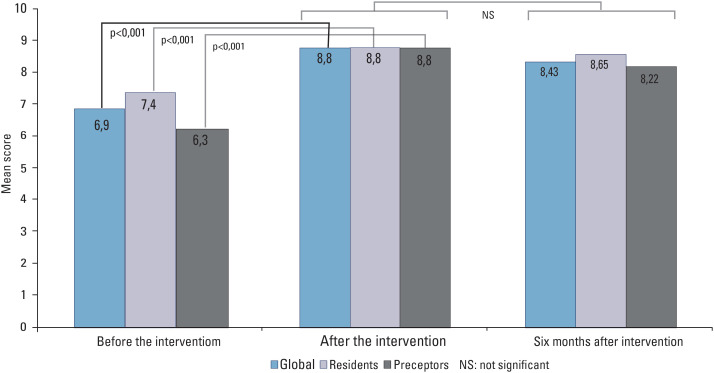
Medical knowledge in hospital hyperglycemia

Medical knowledge improved after the intervention and there was no significant reduction in the rate of correct answers after 6 months (Figure 1).

**Figure 2:**
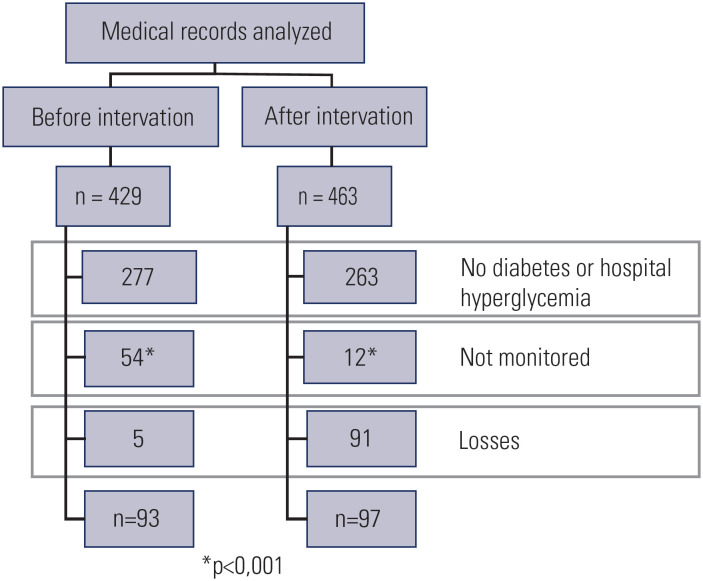
Medical records analyzed before and after the intervention

Regarding the medical records, 429 were reviewed before and 463 after the intervention (Figure 2). The number of patients who did not have at least one glucose assessment during the entire hospitalization for acute illness decreased from 12.6% to 2.6% (p < 0.001). Table 2 describes the characteristics of the patients and the improvement in the patients’ clinical outcomes can be assessed in Table 3. One of the most impressive results of our trial was zero prescriptions with sliding-scale insulin as the main treatment.

**Table 2:** Characteristics of the sample of patients whose medical records were analyzed before and after the intervention

Variables		Before intervention (n = 93)	After intervention (n = 97)	p value
Age (years)		62.8 ± 15.4	64.1 ± 13.3	0.535
Sex (n)		51 (54.8)	48 (49.5)	0.460
Type of DM (n)				0.083
	DM1	5 (5.4)	2 (2.1)	
	DM2	63 (67.7)	79 (81.4)	
	HH	25 (26.9)	16 (16.5)	
Duration of diabetes (years)		10 (2 - 15)	6 (2 - 10)	0.660
HbA1C (%)		8.7 (6.7 - 12.6)	7.3 (6.6 – 10.5)	0.153
Hospitalized for DM (n)		9 (9.7)	3 (3.1)	0.062
Main diagnosis at discharge (n)				
	Cancer	30 (32.3)	28 (28.9)	0.612
	Infection	15 (16.1)	15 (15.5)	0.900
	Covid-19	1 (1.1)	23 (23.3)	<0.001
Presence of comorbidities (n)		86 (92.5)	93 (95.9)	0.315

Data presented as mean ± standard deviation, n (%) and median (interquartile range).

DM: diabetes mellitus; DM1: diabetes mellitus type 1; DM2: diabetes mellitus type 2; HH: hospital hyperglycemia; HbA1C: glycated hemoglobin.

**Table 3:** Hospital outcomes before and after medical education intervention

Variables	Before intervention (n = 93)	After intervention (n = 97)	p value
Metformin use (n)	23 (24.7)	30 (30.9)	0.341
NPH insulin use (n)	31 (33.3)	50 (51.5)	0.011
Regular insulin use (n)	17 (18.3)	5 (5.2)	0.005
Sliding-scale insulin use (n)	2 (2.2)	0 (0)	0.147
HbA1C request (n)	37 (39.5)	51 (52.6)	0.077
Correct monitoring in patients with OR (n)	52 (80)	66 (82.5)	0.701
Correct monitoring in patients with NT (n)	12 (63.2)	12 (70.6)	0.637
Antibiotic therapy (n)	62 (66.7)	64 (66)	0.920
DM expert assessment (n)	10 (10.8)	10 (10.3)	0.921
Hospital stay (days)	15 (9 – 31.5)	13 (7 – 28)	0.529
Capillary glycemia in the target	22.2 (9.1 – 33.3)	22.2 (12.5 – 40.0)	0.588
Hyperglycemia	38.5 (12.9 – 62.5)	45.5 (20.0 – 67.7)	0.312
Hypoglycemia	0 (0 – 1.9)*	0 (0 – 0)*	0.026

Data presented as mean ± standard deviation, n (%) and median (interquartile range)

* Data shown as median (p5 to 95).

NPH: neutral protamine Hagedorn; HbA1C: glycated hemoglobin; OR: oral route; NT: nasoenteral tube; DM: diabetes mellitus.

## DISCUSSION

Our study demonstrated that it was possible to improve medical knowledge on hospital hyperglycemia after a medical education program. Such improvement occurred for both physicians in the residency program and preceptors, which resulted in a 90% correct answer level. This level of knowledge was maintained after 6 months.

We had small error rates even in the pre-test evaluation. It was most likely due to the continued intervention of preceptors specializing in diabetes and hospital hyperglycemia, even prior to the study period in question, thus characterizing a conservative bias. These data highlight the importance of the presence of a specialist conducting active consultancies and continuing medical education within the hospital.

The medical education program on hospital hyperglycemia also led to an improvement in the outcomes of hospitalized patients. After the intervention program, there was not a single prescription of isolated regular insulin in sliding scale given as the main treatment. This shows the benefit of having a medical education program, given that this type of treatment is strongly discouraged (1). There was also an increase in NPH insulin prescription, which is another strong point of this intervention’s results because guidelines suggest that hospital hyperglycemia should be managed with either basal insulin or basal-bolus insulin as the main agents (1,6).

The improvement in medical prescription was most likely associated with the reduced rates of in-hospital hypoglycemia. Hypoglycemia is considered one of the events that needs to be prevented during hospitalization because it is possibly associated with an increase in in-hospital mortality (1,14).

The rates of measurement of blood glucose at least once at admission (as the guidelines recommend) and of glycated hemoglobin for those with hospital hyperglycemia also increased after the intervention, although the latter was not significantly increased. This shows that medical education can improve not only medical prescriptions but also appropriate tests ordering.

Another strong point of our study was the development of a questionnaire for medical knowledge regarding hospital hyperglycemia. It was built based on data from national and international guidelines (1,6,10) on the subject. All questionnaire items were evaluated by a panel of three endocrinologists with research interest, expertise in the management of hospital hyperglycemia, and involvement in medical education and residency programs. The same experts participated in assessing the content and face validity. Moreover, 10 volunteers and different academic areas evaluated the questionnaire in a pilot study regarding the appearance and content to identify ambiguities and lack of clarity. Subsequent studies should assess this questionnaire’s accuracy.

Our study’s limitations were that 1) there was, prior to the study, constant contact of resident physicians with a team of specialists in diabetes and hospital hyperglycemia, as mentioned above, which may have contributed to lower error rates in the questionnaire applied before the intervention (pre-test). 2) The lack of a standardized questionnaire, although this point was minimized by the fact that the authors conducted a pilot study before the study and validation analysis after it. 3) During data collection, we had difficulties in accessing some medical records due to limited access during the COVID-19 pandemic and change in address of the agency responsible for storing them, which led to losses in the post-intervention period. We understand the loss of patients may affect the analysis and conclusion of the results. 4) It is possible there was bias due to the same participants answered the same questionnaires. However, this bias is unlikely. The tests were applied at least one month apart and the participants did not have access to the pre- and post-test; therefore, they did not receive a copy of them. In addition, the benefit was reflected in patients both in the short and long term. 5) The number of losses in the analysis of medical records after the intervention and the mismatch in some of the characteristics of the patients in the groups before and after the intervention can make the comparison inaccurate. 6) The assessment of capillary blood glucose for the 3 days prior to patient discharge, when measuring plasma glucose, which is not possible at that time, is considered a standard method of glycemic assessment.

The strengths of our study were 1) intervention with preceptors and resident physicians, with preceptors from different clinical areas and specialties, ensuring generalizability of the results; 2) good adherence to the intervention, with the participation of almost the entire sample of preceptors and residents of the hospital’s internal medicine department; 3) a large number of medical records from patients from different clinical areas were analyzed, which also increased the external validity of our study; 4) the assessment of knowledge in the short- and moderate/long-term; and 5) constructing a questionnaire specific to the topic.

In conclusion, a medical education program on hospital hyperglycemia at a university hospital improved the knowledge of both residents and preceptor physicians on the subject. The intervention resulted in improved clinical outcomes for hospitalized patients.
